# Evaluating the impact of transmission mode, calibration level and farmer compliance in simulation models of paratuberculosis in dairy herds

**DOI:** 10.1038/s41598-018-27518-7

**Published:** 2018-06-14

**Authors:** Carsten Kirkeby, Kaare Græsbøll, Tariq Halasa

**Affiliations:** National Veterinary Institute, Technical University of Denmark, Kemitorvet, 2800 Lyngby, Denmark

## Abstract

Simulation models can predict the outcome of different strategies for the control and eradication of paratuberculosis (PTB) in dairy herds. Two main transmission modes have previously been used to simulate the spread of PTB: direct (contact between animals) and indirect (through the environment). In addition, previous models were calibrated to either low or high within-herd prevalence levels, which we refer to as normal and low hygiene levels, respectively. We simulated both direct and indirect transmission with the same model in both normal and low hygiene level scenarios. The effectiveness of a test-and-cull strategy was dependent on the calibration level of the simulation model, and eradication occurred less frequently with the more biologically plausible indirect transmission mode. The results were compared to within-herd prevalence records from 314 dairy herds. The prevalence in 50% of the herds varied less than 0.9% per year on average, and less than 4% in 90% of the herds. We therefore conclude that the normal-hygiene scenario best describes most dairy herds in Denmark. Finally, we simulated different levels of farmer compliance with a test-and-cull strategy and found that a 60% compliance level was not sufficient to reach eradication within 10 years.

## Introduction

Paratuberculosis (PTB) is a chronic disease in cattle that is caused by *Mycobacterium avium* ssp. *paratuberculosis* (MAP) and shed predominantly in the feces, but also in milk from infected animals. It is a slowly developing disease that often shows clinical signs late in the life of a cow^1^. Infected cows may exhibit weight loss, reduced milk production and fatal diarrhea. Infection can be transferred horizontally through the milk and feces of other infected animals, with calves being more susceptible to infection than older animals. Calves are also susceptible to vertical transmission (in utero), but we focus on the horizontal transmission in this study.

Simulation models are often used to evaluate the effect of different strategies in controlling diseases^2^. A number of simulation models have been developed for PTB^3^. These models simulate the horizontal spread of MAP via two distinct transmission modes: direct transmission mode (DTM) and indirect transmission mode (ITM). DTM models assume direct transmission of bacteria through contact between infected and uninfected animals in the herd. ITM models assume indirect transmission to uninfected animals through bacteria shed in the environment by infected animals. Models that have used direct horizontal transmission include^4–10^, while models simulating indirect horizontal transmission include a beef herd model by Humphry *et al*.^11^ and four dairy herd models^12–15^. Furthermore, Slater *et al*.^16^ have created a simulation model with both direct and indirect PTB transmission. From a biological point of view, ITM models are more plausible because MAP is transmitted between animals via the environment^1^. However, it is unclear whether results from the DTM and ITM models differ due to the transmission mode or other reasons such as parameterization, population dynamics, etc.

Another feature that separates the currently published PTB simulation models into two groups is the resulting true within-herd prevalence after several years in a no-control situation. Some models predict that the true within-herd prevalence in a no-control scenario will increase to a very high level over time, while some predict more moderate levels (see further description below). We used this simple condition to classify the models in terms of their predicted prevalence. Models that resulted in a high true within-herd prevalence in no-control situations include Collins & Morgan^4^, who predicted the end prevalence after 50 simulated years to be 40–60%. In the model by Groenendaal *et al*.^5^, the predicted true prevalence was 55% after 20 years. Pouillot *et al*.^6^ simulated large herds with a yearly culling rate of 30% and found an incidence rate of about 0.22 after 10 simulated years, corresponding to a true prevalence of around 35%. The model by Kudahl *et al*.^7^ simulated a no-control scenario and predicted a true prevalence of 80% after 10 simulated years. Lu *et al*.^9^ simulated 25 years and predicted a prevalence of 23%, while the models by Marcé *et al*.^14^ and Robins *et al*.^15^ both predicted 88% prevalence after 25 simulated years. Massaro *et al*.^16^ simulated 10 years and predicted a prevalence of 37%, and the model by Al-Mamun *et al*.^12^ resulted in a true prevalence of 49% after 25 simulated years. These models all resulted in a high true prevalence in a no-control scenario, and we can therefore consider that they all reflect low-hygiene scenarios because the true prevalence (and consequently the level of MAP transmission) is closely linked to the hygiene in the herd. Herds with low hygiene levels have a higher potential for MAP transmission because they generally result in a high true prevalence in a no-control situation.

The following models resulted in lower true prevalence in a no-control situation than the models mentioned above, and we therefore consider them to reflect normal-hygiene scenarios. Mitchell *et al*.^8^ showed that the true prevalence could be between 2% and 9% after 25 simulated years, and Smith *et al*.^17^ recently showed that a test-and-cull strategy was appropriate for reducing the true prevalence using the model by Mitchell *et al*.^8^. The resulting true prevalence in a no-control situation was 10–12% after 5–25 years, as shown in Smith *et al*.^17,18^. Kirkeby *et al*.^13^ published a model in which the true prevalence was calibrated to remain around 6% or 45% in order to compare normal- and low-hygiene herds, and as a result, this model reflects both normal- and low-hygiene scenarios. This was also the case for the study by Al-Mamun *et al*.^19^, where calibration resulted in 10%, 23% or 35% true prevalence.

There is clearly a large variation in the predictions of these models, both in terms of the predicted true within-herd prevalence and in the predicted efficacy of control strategies. Very high true within-herd prevalence levels have not previously been seen in Danish dairy herds, and we therefore wanted to investigate the reasons behind the considerable difference in the predicted true within-herd prevalence. The important question that remains is what actually drives these differences - the way they simulate transmission, or the calibration of the models? It is important to understand what prompts these differences in order to understand and interpret model results used to design disease control programs. Furthermore, it is important to analyze how the models reflect real herds, and which models can be used to represent which situations. We focus on test-and-cull scenarios here, because we have previously found this approach to be efficient^13^.

A simulation model is a simplification of real-life systems, and as such, it is expected to detect discrepancies between the predicted scenario and what is seen in reality. One aspect where models can differ from reality is in compliance. For instance, if a farmer does not comply with the recommendations in a control program (for reasons related to perception, behavior and preference), this might have an impact on the effect of the implemented control actions. We therefore also wanted to explore the effect of test-and-cull strategies where farmer compliance is below 100%.

In this study, we aimed to compare direct and indirect transmission modes in the same simulation model of a dairy herd. We simulated a closed herd as these represent 51% of Danish herds and are recommended to prevent the spread of PTB. The purpose of this comparison was to understand why models provide different results and recommendations in terms of test-and-cull strategies. Furthermore, we aimed to compare two scenarios for each transmission mode – normal and low hygiene – in order to evaluate whether calibrating the model to reflect a specific hygiene level on the farm had an impact on the efficiency of the test-and-cull strategy, and to assess whether the normal- or low-hygiene scenario more closely reflected reality in Danish dairy herds. We used a dataset with the apparent within-herd prevalence over time for 314 herds and analyzed trends in the data. Finally, we assessed the impact of farmer compliance on the control of PTB in the herd to ascertain what level of compliance reflected the analyzed data.

## Materials and Methods

### Herd model

We used the iCull model, which simulates a standard Danish dairy herd with 200 lactating cows^13^. It is a stochastic, dynamic bio-economic model that simulates individual animals within a herd. The model has been parameterized by a large dataset of Danish dairy herd data and therefore represents a standard Danish dairy herd where calves are reared into heifers then inseminated, with a given probability of success, and bull calves are sold. After calving, cows go through a lactation period where they have their own lactation curve and somatic cell count (SCC) curve with realistic daily variation. If bulk tank SCC exceeds 200,000 cells/mL, the farmer is penalized^13^. Cows are dried off after the lactation period and the farmer rears all calves, therefore no animals are imported into the herd. This represents 51% of Danish dairy herds with closed systems^20^. Cows are subject to spontaneous mortality (due to diseases other than mastitis) and the farmer culls the excess number of cows in the barn once per week. One third of the culled cows are chosen voluntarily according to their milk production, SCC level and number of previous infections. Two thirds of the culled cows are involuntarily culled, reflecting animals with injuries and diseases other than mastitis. The model is explained in detail in Kirkeby *et al*.^13^, and the input parameters are presented here in the Supplementary Information (Tables S1–S4).

In the simulation model, the probability of exposure to infection, *P*j, is multiplied by the susceptibility measure for each cow, which is a value between 0 and 1, where newborn calves have a susceptibility of 1 and older cows have a susceptibility close to 0. The susceptibility is therefore an exponentially decreasing function, decreasing to 2.6% at the age of 1 year and 0.07% at the age of 2 years^13^. In the model, infected animals first enter a low-shedding state, during which small amounts of MAP are shed. They then enter a high-shedding state, during which larger amounts of MAP are shed. Finally, if they have not already been culled, they will enter the affected state, during which they also shed large amounts of MAP and become clinically ill. Animals are tested four times a year with an ID Screen milk ELISA (IDvet, Grabels, France)^21^. Animals with two or more positive tests out of the last four are identified as being infected. The susceptibility to infection decreases for each animal over time^13^. The test sensitivity in the model is dependent on the age of the tested animal, increasing to about 74% at the age of 5 years^22^. We also simulated scenarios with the sensitivity reduced to 50% and 20% of the original, in order to ensure that our results were robust to changes in this parameter. We did this by reducing the test sensitivity vertically, so it was still dependent on age (Fig. S1, Supplementary Information).

### Indirect transmission mode

The spread of MAP in the original iCull model is via an indirect transmission mode (ITM), where animals are infected through bacteria in the environment^13^. The environment in the simulated housing is divided into five sections: calves, heifers, lactating cows, dry cows and calving area^13^. In brief, the daily probability of exposure to infection from the environment is defined by:

1 $${\rm{Pj}}=1-\exp (\frac{-{\rm{F}}\cdot {\rm{MAPj}}}{H})$$

where *P*j is the probability of each animal being exposed to infectious MAP shed in farm section j; H is the hygiene level on the farm; F is the force of infection parameter, and MAP_j_ is the cumulative amount of MAP shed in section j. The daily amount of MAP shed in each section is subject to a daily exponential survival rate, so that after 385 days only 1% of the shed bacteria will be alive^13^. By dividing this by the hygiene level, H, the probability is balanced so that if the farmer improves the hygiene, fewer infections will occur. Therefore, the higher the hygiene level, the lower the likelihood of transmission. The force of infection was originally calibrated so the true within-herd prevalence in the simulation model was stable over 10 years by adjusting the force of infection parameter. In this study, we used two forces of infection – one that caused a low stable true within-herd prevalence, and one that caused the true within-herd prevalence to increase to a high level. MAP is shed from infected animals, and clinically affected animals that have been infected for a long time will shed more MAP than subclinically affected animals. The surviving MAP was summed up in each section for each simulated day, affecting the probability of exposure for the animals in the section. In addition, MAP can be transferred between farm sections by machinery and personnel^13^, and the hygiene level has no effect on this mode of transfer.

### Direct transmission mode

Most simulation models for PTB within herds use a direct transmission mode (DTM), where disease is transmitted directly by contact between animals, rather than indirectly through the environment (also known as a Reed-Frost model). Therefore, to compare the ITM and DTM models, we also created a version of the iCull model where PTB transmission was based on a contact rate between the animals. The daily probability of an animal being exposed to infectious MAP was then defined by:

2 $${\rm{Pj}}=1-\exp (-{\rm{\beta }}\mathrm{RF}\cdot \frac{{\rm{Ij}}}{{\rm{Nj}}})$$

where *P*j is the probability of exposure to MAP, βRF is the force of infection for MAP, *I*j is the number of infected (subclinical and clinical) animals present in section j, and *N*j is the total number of animals present in section j. Transmission in the DTM model does not include a hygiene parameter, and therefore the force of infection is the only means of adjusting the transmission.

### Calibration

In this study, we used the two transmission modes that simulate the two different scenarios. We calibrated the ITM model by adjusting the hygiene level and evaluated the end prevalence visually. The force of infection works antagonistically with the level of hygiene, so a reduction in hygiene corresponds to an increase in the force of infection. As a result, it was essentially the same parameter that was adjusted in the two models. We calibrated the DTM model by adjusting the force of infection. For each transmission mode, we also tested a normal-hygiene scenario (in which the model was calibrated to maintain a stable true within-herd prevalence) and a low-hygiene scenario (in which the force of infection was adjusted so the true within-herd prevalence would increase to about 80% over 10 simulated years). Although we refer to these as normal- and low-hygiene scenarios, it is possible that different strains with variation in transmission parameters, or different livestock properties like susceptibility to MAP could also have an effect.

We simulated all four combinations of transmission modes and hygiene scenarios in two different situations – first, a no-control situation and secondly, a scenario where a test-and-cull strategy was implemented. Each country has different test strategies, and we used the Danish strategy where cows are tested four times a year with an ID Screen milk ELISA (IDvet, Grabels, France)^20^.

All simulations started with a 6% true within-herd prevalence, which was found to be the median true within-herd prevalence in Danish dairy herds with no control measures against MAP^13^. We ran 500 repetitions of each simulation, simulating 10 years after a 3-year burn-in period. The burn-in period was used to stabilize the model before running each scenario.

### Data

We also wanted to evaluate whether PTB generally showed a trend toward moderate or high within-herd prevalence in dairy herds. To do this, we analyzed a dataset of 314 herds that had participated in the Danish PTB control program, and recorded the apparent within-herd prevalence of PTB-infected cows using a commercial MAP-specific antibody ELISA^23^. The Danish PTB control program started in 2006 and is voluntary. In general, farmers participating in the program must test all lactating cows four times a year and cull cows with two or more positive results from the last four tests^20^. Of the herds in the dataset, 70% (221) had more than 15 records (see Table S5 in Supplementary Information). We used data from 15 October 2008 to 01 January 2014, when most herds had four recordings of the apparent prevalence per year. Seven of the herds had only one recording date in this time period and were therefore omitted from the analysis. We used records from the remaining 307 herds and transformed the apparent prevalence (AP) at each sampling point to true prevalence (TP) with the Rogan-Gladen estimator^24^:

3 $${\rm{TP}}=\frac{{\rm{AP}}+{\rm{Sp}}-1}{{\rm{Sp}}+{\rm{Se}}-1}$$

where Sp is the specificity of the test and Se is the sensitivity of the test. However, the sensitivity of the test is age dependent, which is incorporated in the iCull model, but is not possible to include in the Rogan-Gladen estimator. We therefore used the mean effective sensitivity (MES) calculated for Danish dairy herds^25^ at 60%. The specificity was set to 98.66%^21^. Both sensitivity and specificity were used as fixed values. If the Rogan-Gladen adjustment resulted in a prevalence below zero or above 100%, we set the estimated true prevalence to zero or 100, respectively.

The herds in the dataset were separated into four groups to visually inspect the trend in estimated true within-herd prevalence further. Group 1 contained herds with a maximum estimated true within-herd prevalence below 10%, group 2 contained herds where the maximum estimated true within-herd prevalence was above or equal to 10% and below 20%, group 3 contained herds with a maximum estimated true within-herd prevalence above or equal to 20% and below 30%, and group 4 contained herds with a maximum estimated true within-herd prevalence above or equal to 30%.

To analyze the trend in the estimated true within-herd prevalence on each farm, we fitted linear regression models to the estimated true prevalence records for each farm over time and extracted the estimated slope parameter. We only included herds with at least three points and used the slopes to estimate the annual variation in the estimated true within-herd prevalence. We also separated the herds into groups based on whether their slope was negative or positive, in order to examine the differences between herds with an increasing and decreasing prevalence.

### Compliance

Finally, we ran a series of simulations using the ITM model to evaluate the effect of farmer compliance with recommendations for test-and-cull. We chose to use the ITM model for this because it is biologically more realistic than the DTM model as bacteria have been found to transmit between individuals mainly via the environment^1^. We simulated the compliance scenarios using the normal hygiene level because this best resembles the situation in the majority of Danish dairy herds. Farmer compliance was modelled on a quantitative scale, meaning that the farmer could comply with anything between 0% and 100% of the recommended culling decisions. For instance, if the farmer showed 80% compliance, he/she would follow the recommendation for 80% of the cows marked for culling. The compliance function was only used for cows marked for culling due to PTB, so the cows could still be culled for other reasons. The culling decisions were drawn from a random binomial distribution for every cow using the compliance level, so that the farmer chose not to cull some cows. The compliance series was run on the normal-hygiene scenario with the ITM model.

### Data availability statement

The datasets used can be acquired from the corresponding author upon request.

## Results

### Simulations

**Figure 1:**
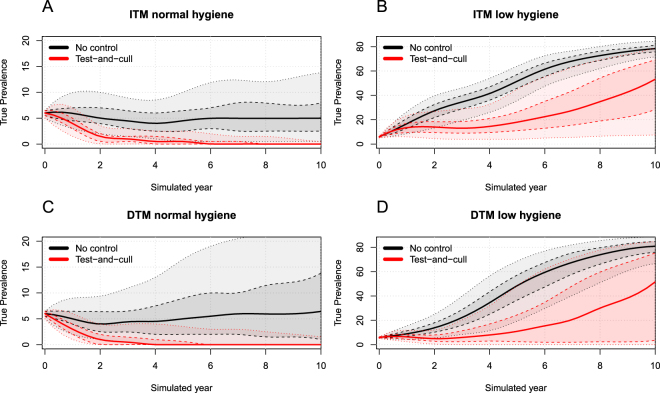
The four combinations of simulated transmission modes and hygiene-level scenarios. (**A**) ITM model with normal hygiene. (**B**) ITM model with low hygiene. (**C**) DTM model with normal hygiene. (**D**) DTM model with low hygiene. Black indicates no control and red indicates test-and-cull. Solid lines represent the median, dashed lines show the smoothed 50% simulation envelope and dotted lines show the smoothed 90% simulation envelope.

**Figure 2:**
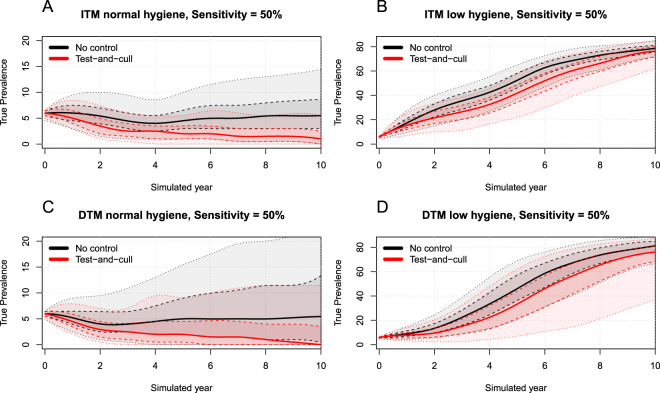
Results of the four combinations of simulated transmission modes and hygiene-level scenarios with sensitivity reduced to 50% of the original. (**A**) ITM model with normal hygiene. (**B**) ITM model with low hygiene. (**C**) DTM model with normal hygiene. (**D**) DTM model with low hygiene. Black indicates no control and red indicates test-and-cull. Solid lines represent the median, dashed lines show the smoothed 50% simulation envelope and dotted lines show the smoothed 90% simulation envelope.

**Figure 3:**
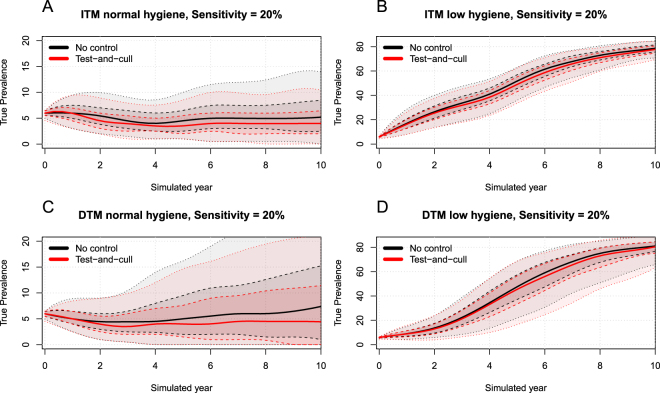
Results of the four combinations of simulated transmission modes and hygiene-level scenarios with sensitivity reduced to 20% of the original. (**A**) ITM model with normal hygiene. (**B**) ITM model with low hygiene. (**C**) DTM model with normal hygiene. (**D**) DTM model with low hygiene. Black indicates no control and red indicates test-and-cull. Solid lines represent the median, dashed lines show the smoothed 50% simulation envelope and dotted lines show the smoothed 90% simulation envelope.

In the normal-hygiene scenarios, the ITM and DTM models showed similar results. In a no-control situation, they both showed a stable true within-herd prevalence around 6% due to their calibration. In a test-and-cull situation, the ITM model was able to eradicate MAP after 6 simulated years (median result, Fig. 1A). The same situation with the DTM model resulted in eradication after 4 years (median result, Fig. 1C). In all test-and-cull simulations, the normal-hygiene scenarios resulted in eradication after a maximum of 8 years (median result). We also found the same patterns in the simulated scenarios with sensitivity reduced to 50% and 20% of the original, but the efficiency of the test-and-cull strategy was reduced (Figs 2 and 3) and eradication could not be achieved within 10 simulated years in the median result for the ITM model with 50% sensitivity. For the DTM model, eradication was reached after 10 years in the median result using 50% sensitivity. Using 20% sensitivity, eradication was not achieved in any of the normal-hygiene scenarios.

In the scenario with the low-hygiene ITM model, a no-control situation resulted in true within-herd prevalence of around 80% after 10 simulated years (Fig. 1B) due to the calibration of the model. Here, a test-and-cull strategy could not eradicate PTB, but only slowed the increase, resulting in a true within-herd prevalence around 50% after 10 simulated years (median). Using the low-hygiene DTM model, the no-control situation likewise resulted in a true within-herd prevalence of around 80% because of the model calibration (Fig. 1D). In this scenario, a test-and-cull situation also resulted in 50% true within-herd prevalence (median), but with a broader distribution compared to the low-hygiene ITM model. Therefore, the test-and-cull situation applied to the DTM model could result in a true prevalence close to zero, even in the low-hygiene scenario (50% and 90% simulation envelopes are shown in Fig. 1). In the simulated low-hygiene scenarios, a sensitivity of 50% only reduced the prevalence by a small amount (Fig. S1). Using 20% sensitivity, eradication was not reached in any of the normal-hygiene scenarios.

The variance in the results was greater for the DTM model than for the ITM model for both low- and normal-hygiene scenarios (Fig. 1).

The annual milk yield and the true within-herd prevalence after 10 simulated years are shown in Table 1. In all four scenarios, the test-and-cull strategy generally resulted in a small increase in the milk yield compared to the no-control scenarios. The milk yield in the normal-hygiene scenarios (A and C) was higher than in the low-hygiene scenarios (B and D). In all simulations, test-and-cull strategies generally resulted in higher milk production than no control, but with a large overlap in their distributions. Furthermore, all test-and-cull strategies resulted in a reduced true prevalence. No formal statistical comparisons of the milk production results were made between scenarios because a statistically significant result can always be achieved by running a sufficiently large number of simulations.

**Table 1:** Summary of the simulated data.

Scenario	Action	ECM 5%	ECM 50%	ECM 95%	TP 5%	TP 50%	TP 95%
(A) ITM: normal hygiene	No control	18.61	**18.84**	19.07	0.49	**5**	13.8
	Test-and-cull	18.66	**18.89**	19.13	0	**0**	0.50
(B) ITM: low hygiene	No control	18.32	**18.56**	18.78	71.6	**78.4**	84.2
	Test-and-cull	18.63	**18.89**	19.17	7.5	**53.2**	79.1
(C) DTM: normal hygiene	No control	18.60	**18.82**	19.07	0	**6.4**	25.7
	Test-and-cull	18.68	**18.90**	19.13	0	**0**	1.52
(D) DTM: low hygiene	No control	18.36	**18.59**	18.85	66.7	**80.9**	88.6
	Test-and-cull	18.63	**18.89**	19.17	0	**51.6**	84.7

The values show the results after 10 simulated years. ECM = annual energy-corrected milk yield after 10 simulated years. TP = the resulting true within-herd prevalence of PTB after 10 years. Values show the median and 5% & 95% simulation envelopes.

### Data

**Figure 4:**
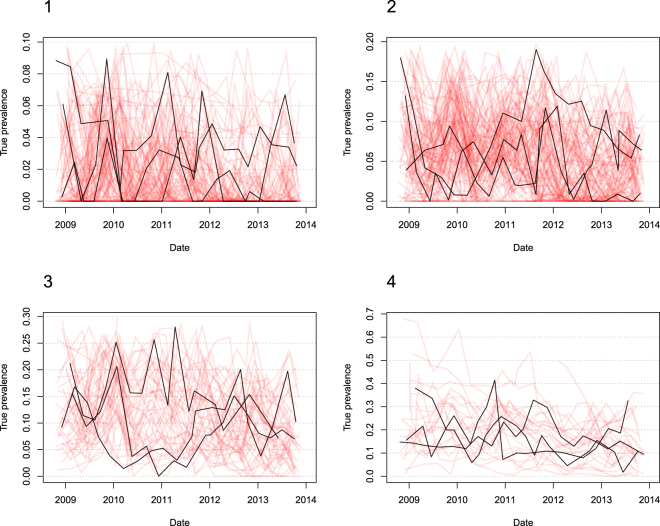
The estimated true within-herd prevalence over time shown for each herd in the field data. The herds are divided into four groups based on the maximum estimated true within-herd prevalence recorded in the herd: group 1 = < 10%, group 2 = 10–20%, group 3 = 20–30%, and group 4 = > 30%. The black lines show records from three randomly selected herds in each group, showing the temporal variation within each herd.

The number of data points from the herds in the field data is shown in Table S5. We analyzed a dataset comprising 307 herds enrolled in the Danish PTB control program to investigate whether PTB generally shows a tendency toward high or low within-herd prevalence. We adjusted the apparent prevalence with the Rogan-Gladen estimator. No herds showed Rogan-Gladen estimates above 100%, and no herds in groups 1–3 had sub-zero estimates in the Rogan-Gladen estimator. In group 4, the herd with most zero estimates in the Rogan-Gladen adjustment had 17 sub-zero estimates. The numbers of zero estimates in this group are shown in Table S6 in the Supplementary Information. Among the 314 herds, 51% had a median estimated true within-herd prevalence below 5%; 80% had a median estimated true within-herd prevalence below 10%, and 90% had a median estimated true within-herd prevalence below 15%. We fitted linear regression models to the estimated true within-herd prevalence records of each herd to analyze the trend over time. In total, 289 herds had three or more data points and were therefore included in this analysis. Of these, 76% (220) had a negative slope and 24% (69) had a positive slope, meaning that most herds showed a negative trend in the estimated true within-herd prevalence over time. The prevalence in half of the herds varied less than 0.9% per year, and 90% of the herds varied less than 4% per year. The herds in the dataset were separated into groups based on their maximum prevalence, as described above. Group 1 (maximum estimated true within-herd prevalence of 10%) contained 113 herds; group 2 (maximum estimated true within-herd prevalence: 10–20%) contained 117 herds, group 3 (maximum estimated true within-herd prevalence: 20–30%) contained 52 herds; group 4 (maximum estimated true within-herd prevalence above 30%) contained 25 herds. The estimated true within-herd prevalence from the field data of individual herds in each group is shown in Fig. 4. By visual inspection, the herds in the first two groups showed an estimated true prevalence that alternated between zero and the maximum estimated true prevalence in the group. There were few recordings of zero in the third and fourth groups. In group 4, 25 herds had a maximum estimated true prevalence higher than 30%, comprising 8% of the herds in the dataset (Fig. 4). The median estimated true prevalence in this group was 17%, the third quartile was 24%, and only two herds had records with estimated true prevalence above 50%. The estimated true prevalence was very rarely stable in any of the herds, but often alternated between relatively low and high. The variance observed was largely consistent with binomial noise, as the sensitivity of test was fairly low and the test specificity was 98.66% (data not shown).

**Figure 5:**
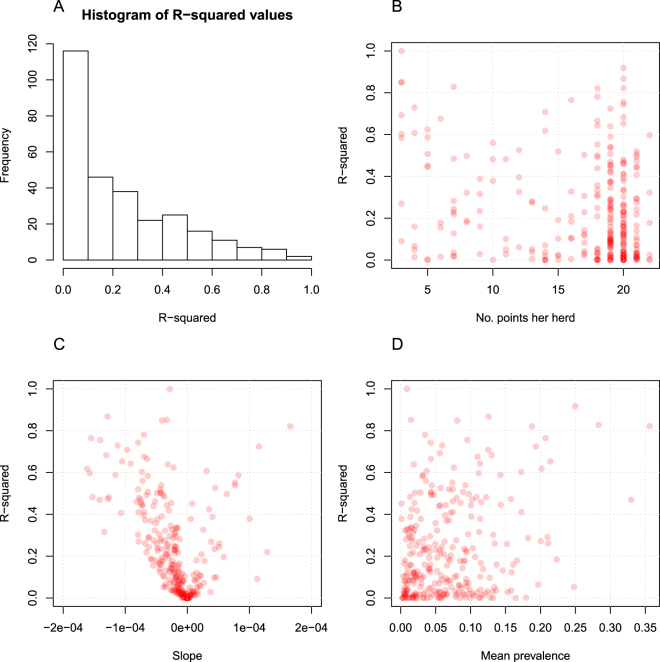
Scatterplots of parameters in the field data. Points are semi-transparent so darker colors represent a higher density of points. (**A**) Histogram of the R-squared values. (**B**) R-squared values for the linear fit as a function of the number of point records per herd, with 20 data points being the most frequent. (**C**) R-squared values for the linear fit as a function of the slope of the regression line for each herd. The X-axis is limited to show the values with near zero slope. (**D**) R-squared values for the linear fit as a function of the mean prevalence in each herd.

Only 15% of the herds had an R-squared value on the slope above 0.5, and 36% of the herds had an R-squared value above 0.25. This indicates a poor general fit in the regression models. Of the herds that had an R-squared value above 0.5, seven had a positive slope and 35 had a negative slope. Of the herds that had an R-squared value above 0.25, 17 had a positive slope and 88 had a negative slope. Figure 5 shows the distribution of R-squared values for the linear regression lines for each herd. It also shows the R-squared values for the linear fit as a function of: the number of point records per herd, the regression line for each herd and the mean prevalence in each herd.

**Figure 6:**
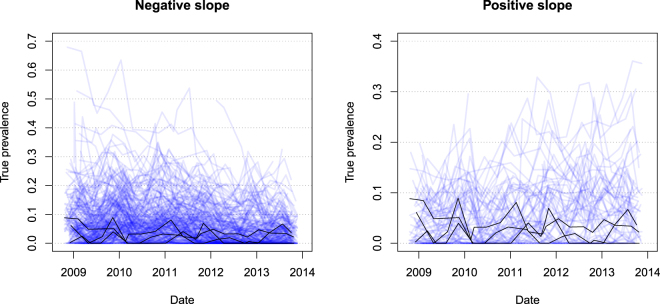
Estimated within-herd prevalence showing a negative slope or a positive slope. Notice that the scales on the y-axes are different. The plots represent 220 herds with a negative slope, and 69 with a positive slope. The black lines show records from three randomly selected herds in each group, showing the temporal variation within each herd.

Figure 6 shows the development of prevalence over time for herds with a general negative slope parameter and a positive slope parameter. There were 220 herds with a negative slope and 69 herds with a positive slope. Most of the herds with a negative slope had a prevalence below 40%, while most of the herds with a positive slope had a prevalence below 30%.

### Compliance

**Figure 7:**
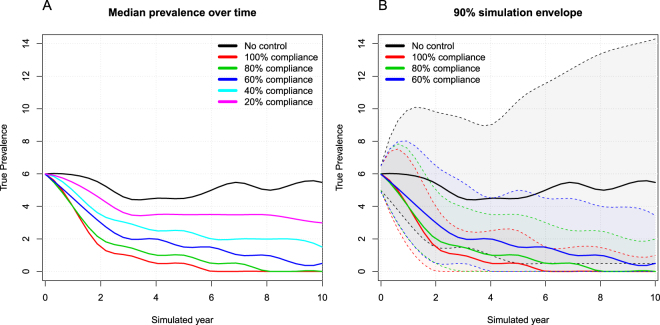
Simulated compliance scenarios. (**A**) Smoothed median true within-herd prevalence over time for the no-control scenario and five different compliance levels. (**B**) Four scenarios are shown with 90% simulation envelopes. No control shows a farmer that does not test-and-cull, 100% compliance simulates culling all of the recommended animals, and 80% and 60% compliance represent the farmer culling 80% and 60% of the recommended animals, respectively. Only the ITM model was used here.

We also simulated a series of compliance situations where the farmer was willing to cull either 100%, 80%, 60%, 40%, 20% or 0% of the cows marked for culling due to PTB (Fig. 7). We used the ITM model and the normal-hygiene scenario for these simulations. In the no-control scenario (0% compliance), the median true within-herd prevalence remained fairly stable at around 6%. In the 100% compliance situation, the median true prevalence dropped from 6% to zero after 6 years, but was still not eradicated after 10 years in all simulations. In the 80% compliance situation, the median true prevalence dropped to zero after 8 simulated years, meaning that MAP would still be present in the herd in half of the simulations. A compliance level of 60% did not result in eradication of PTB within 10 simulated years in the median value (Fig. 7).

## Discussion

The ITM and DTM models yielded similar results for the simulated scenarios in this study, and a test-and-cull strategy was almost equally effective in both models, but with higher variation in the DTM model (Fig. 1, Table 1). The results indicate that the difference between simulation models resulting in very high true prevalence and those with more moderate true prevalence predictions lies in the calibration. The predicted impact of control measures can therefore be affected to a considerable degree by the calibration of the model. Our results show that the test-and-cull strategy is efficient in both the direct and indirect transmission models provided they are calibrated to a stable true prevalence and assuming a high compliance level. In this case, both models indicate that the true prevalence in a no-control situation will remain relatively low. If the simulated transmission is adjusted to reflect a higher level of true prevalence, the impact of a test-and-cull strategy is limited (Fig. 1, Table 1). However, in the DTM model, this strategy could actually keep the true prevalence very low in some of the simulations. This is only possible because test-and-cull is implemented before the true prevalence increases to 80%. To investigate this, we also conducted simulations with a true prevalence starting at 80%, but eradication was not possible in these situations (data not shown). In the ITM model with the low-hygiene scenario, eradication was not possible with the test-and-cull strategy. This is likely to be a result of the latency built into the ITM model, where MAP can survive in the environment even though no animals are infected. These differences between the models become important when evaluating the efficiency of a given control program. Therefore, since ITM models are biologically more plausible and reflect the nature of MAP more realistically, we recommend that ITM models are used for future research. In the present study, we found distinct differences between the normal- and low-hygiene scenarios for both the DTM and ITM models. Test-and-cull was generally successful in the normal-hygiene scenarios, but not in the low-hygiene scenarios. Dairy herd simulation models of MAP can generally be separated into those that reflect normal hygiene and those that reflect low hygiene (calibrated to reflect stable true within-herd prevalence). The low-hygiene models will result in a much higher predicted true within-herd prevalence than the normal-hygiene models. Most importantly, a test-and-cull strategy was generally not successful in eradicating paratuberculosis in the low-hygiene scenarios, whereas it did eradicate the disease in the normal-hygiene scenarios. However, this also depended on the simulated compliance of the farmer (Fig. 5).

The simulated scenarios presented here reflect a closed herd. However, we believe the results can also reflect an open herd, because the purchase of infected animals would not be expected to increase the within-herd prevalence directly to a high degree, but would instead have more of an impact on eradication strategies, as MAP is continually introduced into the environment^25^. In Fig. 5A, the distribution of R-squared values for the linear regression lines for each herd shows a poor fit for many of the regression models. This is likely to be due to fluctuations in the prevalence that can arise from intermittent shedding patterns, or when infected animals are dried off and thus not tested in a sampling round. However, we still found indications that more herds had a negative slope than a positive slope. Of the herds that had an R-squared value above 0.5, seven had a positive slope and 35 had a negative slope, indicating that most herds demonstrating a clear trend have decreasing prevalence. However, the large number of remaining herds (247) with no such trend indicates that conclusions based on these fitted regression models must be drawn with caution. Figure 5B shows the R-squared values for the linear fit as a function of the number of point records per herd. It indicates that most herds have around 20 data points, including herds with either poor or decent model fit. This highlights the variation in the prevalence within each herd, even when many sampling points are available. Figure 5C shows the R-squared values for the linear fit as a function of the slope of the regression line for each herd. It shows that most of the slopes are negative, especially those with high R-squared values. This indicates that the herds with a decrease in prevalence also have less variation in prevalence because the model fit is better, meaning that herds enrolled in the control program generally show a clear decrease in prevalence, with very few showing a clear increase. Furthermore, we found that prevalence in 90% of the herds varied less than 4% per year, indicating that controlling PTB in herds is a marathon rather than a sprint. Finally, Fig. 5D shows the R-squared values for the linear fit as a function of the mean prevalence in each herd. The lack of correlation indicates that the prevalence does not affect the model fit, so that the stability of the estimated prevalence is not associated with either high or low prevalence.

Figure 6 shows that herds with a positive slope, i.e. increasing prevalence, mostly have a relatively low prevalence compared to herds with a negative slope. This supports the findings of the simulation model – that herds with a high prevalence do not generally show an increase in prevalence. Whether this arises from the farmers implementing more control actions as the prevalence increases, or the hygiene level in Danish herds not supporting very high prevalence remains unknown. If the latter is true, the pattern we see in Fig. 6 represents natural fluctuations in prevalence, where herds with low prevalence will have an increasing trend, and herds with high prevalence will have a decreasing trend. It can therefore be seen as a continual regression toward a stable prevalence level, like in the model calibrated to a normal hygiene level.

While simulation models can reflect a simplification of reality, they will never match it perfectly. The estimated true within-herd prevalence in the field data analyzed here was very rarely completely stable in any of the herds, but often alternated between relatively low and high (Fig. 4). This could be influenced by the sensitivity and specificity of the test, cows being dried off (thus changing the total number of cows, and possibly the number of infected cows), or variation in the shedding patterns^16^. Small variations in the apparent within-herd prevalence records would be enhanced by the Rogan-Gladen adjustment. Furthermore, we used the MES as a best estimate of the herd sensitivity for all herds. This will have had an impact on the precision of the result, but it was deemed to be the best option given the available data. Overall, the larger the mean apparent prevalence within a herd, the larger the variation between different time points. This is similar to binomial noise, because the test itself is a binomial process with a positive or negative outcome. Increasing the true prevalence would also increase the variation in the result of this binomial process. In 80% of the herds, the median estimated true prevalence was below 10%. A more general picture could be obtained if we had access to data from herds not enrolled in a control program, thus reflecting no preventive measures. However, our data represent farmers aware of PTB, which must be the main target group for any decision-support tool.

The estimated true within-herd prevalence in 102 herds not enrolled in a control program is presented in Kirkeby *et al*.^13^. Of these, 75% have an estimated true within-herd prevalence below 10%, which is very similar to the 80% found among the 314 herds in the dataset used here. This similarity could reflect farmers demonstrating low compliance with the recommendations of the program. It also supports our hypothesis that the true prevalence within Danish herds generally does not increase considerably if no PTB-specific control actions are implemented, reflecting a normal hygiene level on the farm. Therefore, modelling transmission of paratuberculosis within a herd with a stable low true prevalence is more appropriate than assuming the true prevalence will increase to a high level in a no-control situation, at least in Danish herds. In this study, we did not vary the hygiene or the control actions against PTB spread within the herd during the simulations. It is likely that farmers experiencing an increase in within-herd PTB prevalence will adopt more control actions and increase the hygiene in the herd. However, the speed of these reactions and the amount of control actions will inevitably vary between farms, thus making it difficult to build a model representative of all dairy herds. We therefore chose a simplistic approach by calibrating the model to a stable low true prevalence, reflecting a situation where the within-herd prevalence would not increase by much. This calibration therefore represents situations where the general hygiene on the farm does not allow the within-herd prevalence to increase, and where it follows fluctuations in the within-herd prevalence. It then becomes important to ascertain which model type is the most useful in any given situation. We suggest that the low-hygiene models are suitable for herds facing considerable challenges, e.g. high true prevalence and/or an aggressive strain of PTB. The normal-hygiene models may be more suitable for herds with lower true prevalence, like the vast majority of herds in the field data used in this study (Fig. 4). Herds in the PTB control program are classified in terms of their risk of being infected, and participating farmers are encouraged to purchase animals from low-risk herds. Herds enrolled in the PTB control program have the disease transmission under control, but consistently have infected animals in the herd. This differs from our simulated scenarios, where herds practicing a test-and-cull strategy should be able to reduce the prevalence quickly if the starting prevalence is not very high. This could indicate that MAP persists in environmental reservoirs, perhaps due to low farmer compliance, aggressive strains of MAP and/or new animals continually being introduced. Future research into strain typing and susceptibility to PTB in individual animals may help to determine the most suitable model for specific situations. The success of a test-and-cull strategy is also dependent on the sensitivity of the test used. As presented, the efficiency of test-and-cull was reduced when the sensitivity decreased (Figs 2 and 3). We used published estimates of the sensitivity of the milk ELISA test used in Denmark^21^. However, the tests used differ between countries, and our results might not be applicable to other regions. It was beyond the scope of this study to analyze this further, and we therefore used the Danish standard test-and-cull procedure, in which cows are classified according to their infection status and those with repeated positive tests are marked for culling^13^.

In the compliance analysis, we observed that even when farmers showed 60% compliance, MAP still circulated after 10 years in more than 50% of the simulations. We believe this reflects the reality of stochasticity in dairy herds: in some cases, MAP survives in the herd by chance, even if the farmer implements control actions. This was also apparent from the data presented here, as many herds with a low prevalence did not achieve full eradication, and low compliance could therefore contribute to the situation seen in real herds. Furthermore, we simulated a closed herd, so if the farmer were to buy animals with a risk of infection, this would contribute to keeping MAP circulating within the herd^26^. In the study by Nielsen and Toft^27^, only 155 of 1,081 herds reported a strict compliance with the test-and-cull strategy, culling positive cows before calving. This corresponds to 14% compliance at herd level. In a later study, Nielsen & Kirkeby^28^ found 71% compliance for culling positive cows before calving. However, in both of these studies, many of the cows remained in the herd for a long time after being identified as infected. In addition, other factors such as vertical transmission, individual susceptibility, varying (rather than simply dichotomous) hygiene levels on farms, strain type and transmission capabilities of the pathogen, and the configuration of the farm into sections could have an impact on the result. The infection force in the simulation model also assumes that the true prevalence remains stable in the median result, while in reality it might vary in some herds where the hygiene level might occasionally drop. These uncertainties make it important to investigate these parameters in field studies.

Based on the results of this study, we conclude the following: (1) We were able to calibrate comparable models using either direct or indirect transmission models; they generally showed similar behavior in terms of progress in prevalence, though indirect transmission models are biologically more plausible. (2) The calibration level of the simulation model reflects the variation in true within-herd prevalence over time, as well as the efficiency of the test-and-cull actions. (3) The test sensitivity is important for the effectiveness of a test-and-cull strategy. (4) Discrepancies between field data and simulated studies suggest that other mechanisms (such as the level of compliance with test-and-cull recommendations) play a role in the prevalence of MAP in dairy herds. (4) Simulated results showed that low compliance could result in the same situation as observed in field data.

## Electronic supplementary material

Supplementary information
