# Supplementary material for: Evaluating the impact of transmission mode, calibration level and farmer compliance in simulation models of paratuberculosis in dairy herds
Source: Sci Rep. 2018 Jun 14;8:9100. doi: 10.1038/s41598-018-27518-7 (PMC6002403; doi:10.1038/s41598-018-27518-7)
Supplement: Supplementary file 1 — Supplementary information [file 41598_2018_27518_MOESM1_ESM.pdf]

Supplementary Information for

Evaluating the impact of transmission mode, calibration level and farmer compliance in simulation models of paratuberculosis in dairy herds

Carsten Kirkeby[1*], Kaare Græsbøll[1], Tariq Halasa[1]

[1] National Veterinary Institute, Technical University of Denmark, Kemitorvet, 2800 Lyngby, Denmark

[*] Correspondence to: ckir@vet.dtu.dk

Table S1. Probabilities, durations of periods and prices used in the model. Durations (except for calfhood) are described by $N(x, sd)$, a normal distribution around the mean x with the standard deviation sd. All prices are in EUR. This table is duplicated from Kirkeby et al. [13].

Parameter	Default value	Explanation	Reference
Heat Detection (Cow)	0.36	Probability of detecting heat (cows) (per heat period)	(Ancker et al., 2009)
Pregnancy Probability (Cow)	0.42	Probability of pregnancy (cows) (per insemination)	(Ancker et al., 2009)
Heat Detection (Heifer)	0.60	Probability of detecting heat (heifers) (per heat period)	(Ancker et al., 2009)
Pregnancy Probability (Heifer)	0.55	Probability of pregnancy (heifers) (per insemination)	(Ancker et al., 2009)
Days Calf	365	Number of days spent as a calf	Expert opinion
Days Heifer	*N* (110, 2)	Number of days spent as a heifer	Expert opinion
Days Inseminated Heifer	*N* (41, 2)	Number of days to spend as inseminated heifer	Expert opinion: Pregnancy can be tested after 41 days.
Days Pregnancy Heifer	*N* (280, 2)	Number of days to spend as pregnant heifer	Expert opinion
Days Milk Cow	*N* (21, 2)	Number of days to spend as a lactating cow between inseminations	Expert opinion
Days Post Calving	*N* (40, 2)	Number of days spent as a cow between calving and insemination	Expert opinion
Days Inseminated Cow	*N* (41, 2)	Number of days spent as inseminated cow	Expert opinion: Pregnancy can be tested after 41 days.
Days Pregnancy Cow	*N* (224, 2)	Number of days spent as pregnant cow	Expert opinion
Days Dry Cow	*N* (56, 2)	Number of days spent as dry cow	www.landbrugsinfo.dk
Max Age	3650	Maximum age before a cow is culled	Expert opinion
Price ECM	0.313	The income (EUR) of selling 1 kg ECM (mean of high and low milk price)	(Aes, 2009)
Feed Unit Cost	0.133	The cost (EUR) for one feed unit (Roughage)	(Aes, 2009)
EUR per Hour	16	The cost (EUR) for one hour labor	(Kudahl et al., 2011)
Insemination Price	16.1	The cost (EUR) for one insemination	(Kudahl et al., 2007b)
Destruction Price	64.8	The cost (EUR) for destroying one animal (Excl. VAT)	www.daka.dk (07/08/2014)
ELISA price	5.3	The cost (EUR) of one ELISA	(Kudahl et al., 2007b)

Table S2. MAP-related parameters used in the simulation model. The columns show each parameter, the default value used in the simulations, an explanation of the implementation of the parameter, and the reference for the chosen value. *N* (*x, sd*) describes a normal distribution around the mean *x* with the standard deviation *sd*. Parameters names and default values used in the iCull model are described and explained. References are given in the right column. This table is duplicated from Kirkeby et al. [13].

Parameter	Default value	Explanation	Reference
Number of Farming machines	1	Number of machines used in all farm sections	
Number of Personnel	2	Number of personnel working in all farm sections	
Machine cross-contamination	8%	The amount of MAP shed within each farm section that is spread with machines	Expert opinion
Boot cross-contamination	1%	The amount of MAP shed within each farm section that is spread with boots	Expert opinion
Force of Infection	0.00016	The force of infection from environmental MAP (scaling parameter)	Calibrated in the model
Hygiene Level	1	The hygiene level on the farm. Lower hygiene level will increase the infection pressure from environmental MAP (scaling parameter)	Default set to 1
Low Shedding	5%	Level of shedding from infected and low shedding animals	Expert opinion
High Shedding	20%	Level of shedding from infected and high shedding animals	Expert opinion
Affected Shedding	100%	Level of shedding from infected and affected animals	Expert opinion
Colostrum Risk	2%	Daily risk of calf getting infected from colostrum from if not pasteurized (per calving)	Calibrated in the model
Wastemilk Risk	0.32%	Daily risk of calf getting infected from wastemilk if not pasteurized (per calving)	Calibrated in the model
Calf Risk 1	9%	Risk of calf getting infected from infected and low shedding mother (per calving)	(Whittington and Windsor., 2009)
Calf Risk 2	9%	Risk of calf getting infected from infected and high shedding mother (per calving)	(Whittington and Windsor., 2009)
Calf Risk 3	39%	Risk of calf getting infected from infected and affected mother (per calving)	(Whittington and Windsor, 2009)
Days State 3	*N* (1095, 109.5)	Number of days spent in the affected state if not culled. Affected cows are rendered after dying from disease	Expert opinion
Days State 2	*N* (365, 36.5)	Number of days spent in the high shed- ding state before being affected	Expert opinion
Days State 1	*N* (180, 18)	Number of days spent in the low shed- ding state before progressing to the high shedding state	Expert opinion
Cutoff	≥ 0.3	Cutoff used to identify a positive ELISA	ID Screen (IDvet, Graebels, France)

Table S3. Initial properties of the simulated herd in the iCull model, showing the distribution of animals in each life stage in the initial herd. Par columns show the percentages of cows in parity 1, 2 and 3+, for each of the life steps. The initial herd is constructed to reflect a medium sized dairy herd with 200 cows. This table is duplicated from Kirkeby et al. [13].

Parameter	Distribution	No. animals	Par 1	Par 2	Par 3+
Calves	27%	118			
Heifers	10%	44			
Inseminated heifers	1%	4			
Pregnant heifers	18%	79			
Early lactation cows	7%	30	57%	30%	13%
Inseminated cows	7%	30	30%	43%	27%
Pregnant cows	25%	110	43%	26%	31%
Dry cows	7%	30	53%	23%	24%

Table S4. Mortalities and stillbirth rates used in the model. This table is duplicated from Kirkeby et al. [13].

Parameter	Default value	Explanation	Reference
death0	0.065	Yearly mortality for calves	Expert opinion
death1	0.035	Yearly mortality for heifers	Estimated to be between 3.2 and 3.7% (SEGES 2015)
death2	0.05	Yearly mortality for inseminated heifers	Expert opinion
death3	0.05	Yearly mortality for pregnant heifers	Expert opinion
death4	0.05	Yearly mortality for milking cows	Expert opinion
death5	0.05	Yearly mortality for inseminated cows	Expert opinion
death6	0.05	Yearly mortality for pregnant cows	Expert opinion
death7	0.05	Yearly mortality for dry cows	Expert opinion
Stillbirth	0.04	Risk of stillbirth per calving	Expert opinion

Table S5. The Numbers of herds with one to 22 records in the field data.

No. of records	No. Of herds
3	8
4	6
5	7
6	4
7	10
8	4
9	5
10	4
11	4
12	4
13	5
14	9
15	6
16	7
17	10
18	25
19	62
20	71
21	32
22	6

Table S6. Number of zero-estimates in the prevalence adjusted with the Rogan-Gladen estimator. All herds are in group 4.

No. of sub-zero estimates in the Rogan-Gladen estimator	No. Of herds
0	23
1	31
2	28
3	9
4	17
5	12
6	8
7	7
8	9
9	14
10	10
11	9
12	6
13	3
15	1
16	2
17	1

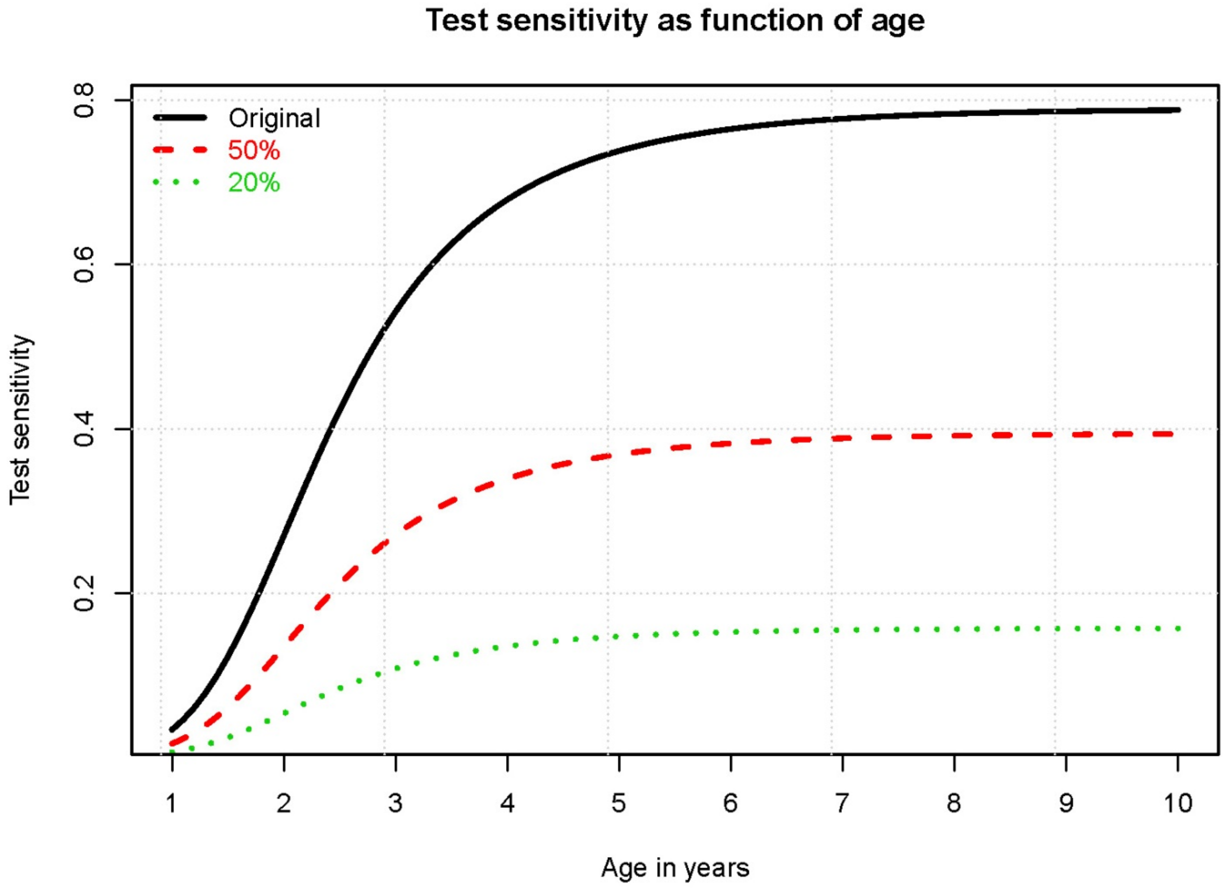

Figure S1. The test sensitivity as a function of the age of the tested animal. This figure shows the original used test sensitivity in the simulation model and the reduced versions at 50% and 20%.
